# M. Bouchardat’s Hint for the Treatment of Diabetes Mellitus

**Published:** 1842-06-18

**Authors:** 


*Hint for the treatment of Diabetes Mellitus.*—M. *Bouchardat*, in a memoir recently communicated to the Academy, remarked that the matter of perspiration is usually found to have lost its normal acid properties, while on the other hand the secretion from the mucous membrane of the digestive canal, which is alkaline in health, becomes acid. He strongly recommends brisk friction of the surface, with the use of warm flannel clothing, and the internal administration of carbonate of ammonia, to restore the healthy state of the cutaneous and mucous secretions.

*Remark.*—That diabetes mellitus is essentially a humoral disease, or in other words dependent upon or connected with an altered state of the fluids of the body, there cannot be a doubt; and it is therefore highly probable that more light may be thrown on its real nature by the discoveries of organic chemistry than by any other mode of examination. Whether M. *Bouchardat* is correct in stating that the cutaneous secretion changes from acid to alkaline, and the mucous secretion from alkaline to acid, we are not prepared to say: we must wait the results of future experiments.

Dr. *Willis*, in his recent work on Urinary Diseases, alludes particularly to the good effects of magnesia in the treatment of many cases of diabetes mellitus, and other practitioners have had occasion to make a similar remark. Perhaps, however, this need not surprise us, considering that, whenever there is any disturbance of the digestive functions, there is always a greater or less tendency to acidity of the stomach. No doubt the use of an animal diet must powerfully contribute to obviate this tendency whenever it exists.—*Medico-Chirurgical Review.* April, 1842.

[In one of the cases published in the Clinical Reports the carbonate of ammonia seemed to act favourably in diabetes.]

